# A Field Study in Benin to Investigate the Role of Mosquitoes and Other Flying Insects in the Ecology of *Mycobacterium ulcerans*

**DOI:** 10.1371/journal.pntd.0003941

**Published:** 2015-07-21

**Authors:** Barnabas Zogo, Armel Djenontin, Kevin Carolan, Jeremy Babonneau, Jean-François Guegan, Sara Eyangoh, Estelle Marion

**Affiliations:** 1 IRD-CREC Cotonou, Bénin, and University of Abomey-Calavi, Cotonou, Bénin; 2 UMR MIVEGEC, CNRS, IRD, Universities of Montpellier I and II, Montpellier, France; 3 ATOMycA, Inserm Avenir Team, CRCNA, Inserm U892, 6299 CNRS, University and CHU of Angers, Angers, France; 4 Laboratoire de Mycobactériologie, Centre Pasteur du Cameroun, Yaoundé, Cameroun; 5 Centre de Diagnostic et de Traitement de l’ulcère de Buruli, Fondation Raoul Follereau, Pobè, Bénin; Fondation Raoul Follereau, FRANCE

## Abstract

**Background:**

Buruli ulcer, the third mycobacterial disease after tuberculosis and leprosy, is caused by the environmental mycobacterium *M*. *ulcerans*. There is at present no clear understanding of the exact mode(s) of transmission of *M*. *ulcerans*. Populations affected by Buruli ulcer are those living close to humid and swampy zones. The disease is associated with the creation or the extension of swampy areas, such as construction of dams or lakes for the development of agriculture. Currently, it is supposed that insects (water bugs and mosquitoes) are host and vector of *M*. *ulcerans*. The role of water bugs was clearly demonstrated by several experimental and environmental studies. However, no definitive conclusion can yet be drawn concerning the precise importance of this route of transmission. Concerning the mosquitoes, DNA was detected only in mosquitoes collected in Australia, and their role as host/vector was never studied by experimental approaches. Surprisingly, no specific study was conducted in Africa. In this context, the objective of this study was to investigate the role of mosquitoes (larvae and adults) and other flying insects in ecology of *M*. *ulcerans*. This study was conducted in a highly endemic area of Benin.

**Methodology/Principal Findings:**

Mosquitoes (adults and larvae) were collected over one year, in Buruli ulcer endemic in Benin. In parallel, to monitor the presence of *M*. *ulcerans* in environment, aquatic insects were sampled. QPCR was used to detected *M*. *ulcerans* DNA. DNA of *M*. *ulcerans* was detected in around 8.7% of aquatic insects but never in mosquitoes (larvae or adults) or in other flying insects.

**Conclusion/Significance:**

This study suggested that the mosquitoes don't play a pivotal role in the ecology and transmission of *M*. *ulcerans* in the studied endemic areas. However, the role of mosquitoes cannot be excluded and, we can reasonably suppose that several routes of transmission of *M*. *ulcerans* are possible through the world.

## Author Summary

Buruli ulcer is a neglected tropical disease due to *M*. *ulcerans*, an environmental mycobacteria. Modes of transmission to human remain unclear and water bugs and mosquitoes had been incriminated with more or less experimental laboratory evidences and filed studies. In this context, we have investigated the presence of *M*. *ulcerans* DNA in mosquitoes and other flying insect in a highly endemic area of Buruli ulcer in Benin. No trace of the bacteria was found in mosquitoes and other flying insects, while 8,7% of aquatic insects, including water bugs, caught in the same area and in the same period were found positive to *M*. *ulcerans* DNA. Our results support the hypothesis that mosquitoes don’t play a major role in ecology of *M*. *ulcerans* in our research area and is in favor of a transmission from the aquatic environment.

## Introduction

Buruli ulcer, which is caused by *M*. *ulcerans*, is a neglected tropical disease affecting mostly poor rural communities in West and Central Africa. In 2013, 75% of all new cases of Buruli ulcer worldwide were declared by Ivory Coast, Ghana and Benin. This skin disease, which mostly affects children, causes large ulcerative lesions often leading to permanent disabilities [1,2,3]. The cutaneous lesions are caused by a *M*. *ulcerans* toxin called mycolactone with cytotoxic, immunomodulatory and analgesic effects [4]. At early stages, Buruli ulcer can be treated with a combination of streptomycin and rifampin for eight weeks; at later stages, antibiotic therapy is associated with extensive surgery [5,6,7,8].

Buruli ulcer occurs mostly in low-lying swampy areas [9,10]. Epidemiological studies have shown that the aquatic environment is the main reservoir of *M*. *ulcerans*, with many aquatic vertebrates and macro-invertebrates harboring this bacillus. The exact ecological features and mode of transmission of *M*. *ulcerans* to humans remain to be identified. In recent decades, several studies have suggested that water bugs and mosquitoes may play a role in *M*. *ulcerans* transmission [11,12,13,14,15,16,17,18,19,20,21,22,23,24,25]. Water bugs have been implicated as potential hosts and vectors of the bacillus in laboratory experiments and field ecology studies in Africa [26,27,28,29,30]. Outside the aquatic environment, adult mosquitoes tested positive for *M*. *ulcerans* DNA in an area of endemic Buruli ulcer in Australia, leading to the suggestion that these insects might transmit the bacterium to humans [26,28,29,30]. However, this hypothesis was not confirmed by laboratory experiments, and, surprisingly, no study has investigated the possible involvement of mosquitoes in *M*. *ulcerans* ecology in Africa, the continent with the highest level of endemicity for Buruli ulcer.

The objective of this study was to investigate the presence of *M*. *ulcerans* DNA in flying insects, including mosquitoes, in an area of Buruli ulcer endemicity in Benin. We monitored, in parallel, the levels of *M*. *ulcerans* in the aquatic environment, as a marker of the presence of the bacterium in the study area.

## Materials and Methods

### Study area

**Fig 1:**
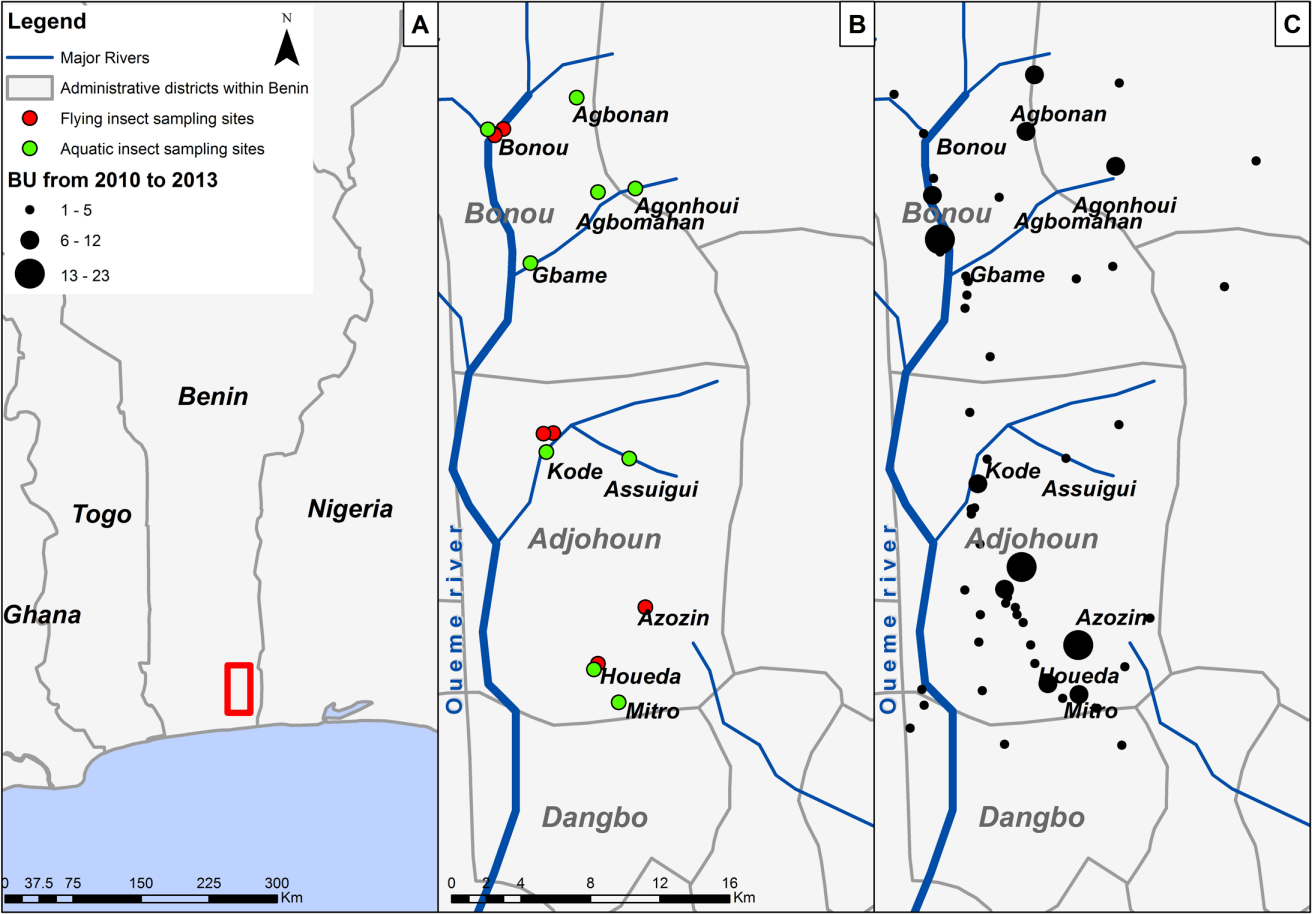
Location of the sites used for environmental sampling in the area of Buruli ulcer endemicity in the Oueme Valley in Benin. A. Area of Buruli ulcer endemicity in the Oueme Valley in South-East Benin (red rectangle). B. Map indicating the sites at which flying insects and larvae were collected (red points) and aquatic sampling was carried out (green points). C. Map showing the locations along the Oueme River of Buruli ulcer patients, from 2010 to 2013 (cumulative cases in black points).

The study was carried out in the Oueme administrative area in South-East Benin, where Buruli ulcer has been endemic for several decades [31,32,33,34,35,36]. Sampling was carried out in three districts crossed by the Oueme River (Bonou, Adjohoun and Dangbo). The districts were selected for study because they are accessible throughout the year (including the rainy season) and because data were available for relevant epidemiological studies. Flying insects were sampled at four sites and aquatic sampling was carried out at nine sites (Fig 1).

The Oueme River originates in the Taneka hills in the Atacora Mountains and flows into the Atlantic Ocean close to Cotonou. The study area is characterized by a subequatorial climate with two rainy seasons. The first rainy season extends from April to July and the second extends from October to November. Mean annual precipitation is 1122 mm, and temperatures range from 22°C to 26°C. There are two main types of soil: alluvial soils, which are fertile but liable to flooding, and sandy soils, which are less fertile but suitable for growing coconut, palm, and other tropical trees. Most of the population in this area is engaged in farming (rice, maize, cassava, cowpeas, market garden crops, etc.), fishing and trade. The natural vegetation consists of grassy savannah and swampy mangrove forest.

### Flying insect sampling

**Fig 2:**
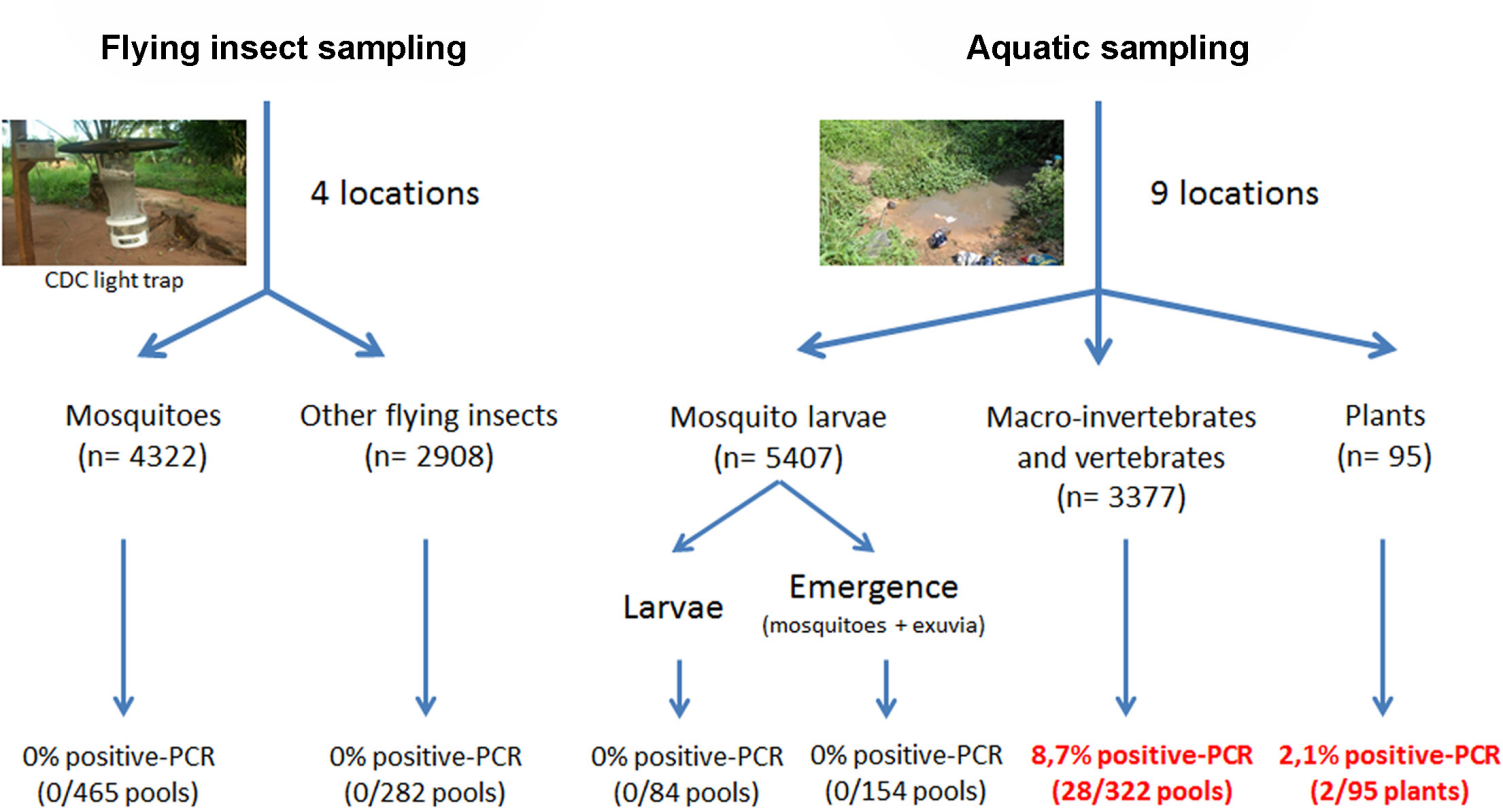
Schematic summary of the environmental sampling and detection of *M*. *ulcerans* by PCR.

This study focused on the adult stage of mosquitoes and other flying insects and the immature stages of mosquitoes. Insects were collected during four surveys in June, July, November, and December 2013, at four sites in the Bonou Centre, Kode, Gbada and Houeda areas (Fig 1). The collection periods correspond to the start, middle and end of the rainy season and the dry season, respectively. Flying insects were collected with Centers for Disease Control (CDC) light traps. A CDC light trap consists of a 150 mA incandescent light bulb and a fan, powered by 6 V batteries. At each survey, once consent had been received from the heads of household, insects were trapped from two selected houses in each village, over a period of two days. Traps were placed both indoors and outdoors at each house, from 6:00 pm to 6:00 am, corresponding to the period from dusk to dawn. The indoor traps were suspended from the ceiling, about 2m above the ground. The outdoor traps were hung on trees at about the same height. The insects collected were identified in the field in two steps. In the first step, mosquitoes were separated from the other insects. All mosquitoes were identified to species level under stereoscopic microscopes, according to morphological criteria in dichotomous keys [37,38,39]. They were counted and stored, in pooled groups of up to 15 individuals of the same species, in 70% ethanol for transport to the laboratory. In the second step, the remaining flying insects were identified to order level on the basis of their morphology under a stereoscopic microscope, with the appropriate keys [40,41]. They were stored in 70% ethanol, in pooled groups of up to 15 individuals from the same order, and were transported to the laboratory for PCR analysis (Fig 2).

### Sampling of mosquito larvae

During each survey, mosquito larvae were collected throughout the selected area by dipping with a 350 ml ladle. Samples were collected from various temporary and permanent bodies of water constituting potential habitats for the development of populations of mosquito larvae. All larvae were transported in clean water, in plastic containers, to the field laboratory. Larvae were identified to genus level with appropriate morphological keys [37,38,39]. The larvae of each genus were then separated into two groups. The larvae of the first group were preserved in 70% ethanol, in pools of 20 individuals for each genus. The larvae of the second group were reared to emergence. The resulting adults were then stored in 70% ethanol, in pools of up to 15 individuals. Exuviae were also preserved in 70% ethanol, in pools of 20, for laboratory analysis (Fig 2).

### Aquatic sampling

Samples were collected from the principal sources of water for domestic washing, bathing, fishing and recreation. The sampling sites were located in nine villages in the three districts: Bonou Centre, Agbonan, Agbomahan, Agonhoui, Gbame, Kode, Assigui, Houeda, and Mitro (Fig 1). Aquatic sampling was carried out with the same methods at each site, at least twice, between January 2013 and December 2013. Invertebrates and fish were captured with a square net (32 x 32 cm and 1 mm in mesh size), from the surface down to a depth of 0.2 to 1 m, over a distance of 1 m. A sample was considered to correspond to all the insects collected in 10 such sweeps with the net. All insects were preserved in 70% ethanol for laboratory identification. For the detection of *M*. *ulcerans* DNA, the insects were sorted into pooled groups, each including no more than 20 specimens from the same family. For each body of water, we collected plant samples from the predominant and the second most frequent types of living plant. Each of these plant samples consisted of one to five plant leaves, stems or roots, depending on the size of the plant sample. They were placed directly in a clean 100 ml bottle containing 70% ethanol (Fig 2).

### Extraction and purification of DNA

Pooled insect bodies were ground and homogenized in 50 mM NaOH. Tissue homogenates were heated at 95°C for 20 min. The samples were neutralized with 100 mM Tris-HCl, pH 8.0. DNA was extracted from the homogenized insect tissues with the QIAquick PCR purification kit (Qiagen), according to the manufacturer’s recommendations. Negative extraction and purification controls were included in each series of manipulations. The homogenizers were decontaminated by incubation overnight in 1 M NaOH, to eliminate any traces of DNA. For each aquatic plant sample, the material was cut into small pieces with a scalpel and then ground in 50 mM NaOH. The extract was heated and neutralized and the DNA was purified with the Mobio purification kit, according to the manufacturer’s recommendations.

### Quantitative PCR

Oligonucleotide primer and TaqMan probe sequences were used for detection of the IS*2404* sequence and the ketoreductase B (KR) domain of the mycolactone polyketide synthase (mls) gene from the plasmid pMUM001 [13,42,43]. PCR mixtures contained 5 μl of template DNA, 0.3 μM of each primer, 0.25 μM probe, and Brilliant QPCR Master Mix (Agilent Technologies) in a total volume of 25 μl. Amplification and detection were performed with a Thermocycler StepOne (Applied Biosystems), using the following program: heating at 95°C for 10 min, followed by 40 cycles of 95°C for 15 s and 60°C for 1 min. DNA extracts were tested at least in duplicates, and negative controls were included in each assay. Quantitative readout assays were set up, based on an external standard curve generated with five tenfold serial dilutions of *M*. *ulcerans* (strain 1G897) DNA. Samples were considered positive only if both the IS*2404* sequence and the gene sequence encoding the ketoreductase B domain (KR) were detected, with threshold cycle (Ct) values strictly < 35 cycles. An inhibition control was performed as previously described [44] and 10% negative controls (water alone) were included in each assay.

### Data analysis

Mosquito abundance was compared between sites and between seasons in nonparametric Kruskal–Wallis tests.

## Results

### Diversity of flying insect orders collected

We collected 7230 flying insects from nine orders: Coleoptera, Diptera, Heteroptera, Homoptera, Hymenoptera, Lepidoptera, Nevroptera, Odonate, Tricoptera. At all sites, Diptera was by far the most frequent order of flying insects caught, accounting for 84% of all insects trapped. Heteroptera was the least abundant order at each site and was not detected at Gbada and Houeda (Table 1).

**Table 1:** Orders of flying insects collected during the surveys.

	Gbada	Bonou	Houeda	Kode	Total
Coleoptera	37	31	16	59	143
Diptera	1136	1033	1822	2056	6047
Heteroptera	0	1	0	1	2
Homoptera	29	5	10	6	50
Hymenoptera	11	71	2	39	123
Lepidoptera	85	41	138	98	362
Nevroptera	54	53	28	67	202
Odonates	2	2	2	2	8
Tricoptera	78	86	31	98	293
Total	1432	1323	2049	2426	7230

### Diversity of mosquito species collected

The 6047 dipteran specimens collected during the four surveys included 4322 mosquitoes from 10 species. *Mansonia africana* (50%), *Culex nebulosus* (27%), and *Culex quinquefasciatus* (22%) were the most abundant species, accounting for 98% of all the mosquitoes trapped. The four least represented species were *Anopheles pharoensis*, *Aedes vittatus*, *Culex decens*, and *Culex fatigans*, with no more than four individuals each (S1 Table).

### Spatio-seasonal variation of the total mosquitoes and flying insects collected

No significant differences in the abundance of the mosquitoes and other flying insects caught were found between sites (*p*>0.05). Flying insects were significantly more abundant (*p*<0.05) in the wet season than in the dry season, whereas no significant difference in mosquito densities was observed within seasons (*p*>0.05; S2 Table).

### Collection of larvae

During the surveys, we collected a total of 5407 mosquito larvae. These larvae were identified as *Culex* spp., *Anopheles* spp. and *Aedes spp*. In total, 3146 mosquito larvae belonged to the genera *Culex* and *Anopheles*. *Culex* spp. were the most abundant, accounting for 66.35% of the mosquito larvae collected. For the adults emerging in the laboratory following the rearing of larvae collected in the field, 2261 individuals belonging to the genera *Culex*, *Anopheles* and *Aedes* were identified. *Culex* was the most abundant genus, accounting for 79.08% of the sample (S3 Table).

### Diversity of aquatic sampling

During the survey, we collected 3377 aquatic vertebrates and macro-invertebrates from various bodies of water in the Oueme administrative area (Table 2). Insecta accounted for 72% of the animals collected, with a majority of Hemiptera. The bodies of water studied were of various natures (flooded land, river and swamp) and were scattered around the Oueme, making it possible to sample diverse types of specimens from different ecological niches. In total, 95 plants were collected from the various bodies of water. They were identified as belonging to the Poaceae, Lemnaceae, Nymphaeaceae, Araceae and Potamogetonaceae families.

**Table 2:** Presence of *M*. *ulcerans* in aquatics vertebrates and macro-invertebrates collected in different areas of Oueme.

			Mitro	Houeda	Agonhoui	Agbomahan	Agbonan	Assigui	Kode	Gbame	Bonou	Total
Vertebrates	Fish		17 (0/5)	1 (0/1)	54 (0/6)	-	2 (0/2)	88 (1/5)	3 (0/1)	3 (0/2)	-	169 (1/22)
	Anura		6 (0/4)	12 (1/4)	19 (0/4)	14 (0/2)	79 (0/3)	18 (1/3)	2 (0/1)	2 (0/1)	12 (0/1)	164 (2/24)
Invertebrates	Insecta	Odonata	36 (0/5)	5 (0/3)	17 (1/4)	-	44 (0/4)	10 (0/4)	2 (0/3)	6 (0/2)	22 (0/1)	142 (1/26)
		Hemiptera	676 (2/36)	216 (1/23)	22 (2/12)	6 (0/3)	269 (1/17)	59 (3/13)	111 (1/15)	122 (0/15)	109 (0/7)	1590 (10/141)
		Coleoptera	288 (2/17)	118 (2/8)	1 (0/1)	-	6 (0/2)	32 (0/5)	75 (1/9)	53 (0/7)	27 (1/4)	600 (6/53)
		Diptera	-	-	-	-	100 (1/2)	-	-	1 (0/1)	1 (0/1)	102 (1/4)
	Mollusca		100 (0/4)	16 (0/2)	148 (0/6)	-	-	1 (0/1)	3 (0/1)	-	-	268 (0/14)
	Crustacea	Decapoda	5 (0/1)	-	93 (2/9)	59 (0/5)	-	150 (5/11)	1 (0/1)	-	-	308 (7/27)
		Araneae	7 (0/1)	3 (0/2)	18 (0/4)	-	1 (0/1)	2 (0/1)	3 (0/1)	1 (0/1)	-	35 (0/11)
		Total	1135 (4/73)	371 (4/43)	372 (5/46)	79 (0/10)	501 (2/31)	360 (10/40)	200 (2/32)	189 (0/29)	171 (1/14)	3378 (28/322)

The figures correspond to the abundance and the figures in brackets correspond to the number of positive qPCR pools (number of positive pools/total pools). qPCR targeting the KR and IS*2404* sequences was used to detect *M*. *ulcerans*. Only sample pools testing positive for both sequences were considered positive.

### Detection of *M*. *ulcerans* DNA in environmental samples

We tested flying insects, larvae, aquatic vertebrates and invertebrates, and plants collected in 2013 from various sites in Oueme for the presence of *M*. *ulcerans* DNA. We found that 942 pools of flying insects (corresponding to the 7230 captured flying insects and the 5407 collected larvae) tested negative for *M*. *ulcerans* DNA by PCR. Positive PCR results were obtained for 8.7% (28/322) of aquatic animal sample pools from the various bodies of water. No positive specimens were obtained at two sites, and 5.5 to 25% of the sample pools at the other seven sites tested positive (Table 2 and S4 Table). Decapoda was the invertebrate family with the highest level of mycobacterial contamination (26%). We performed 295 PCR analyses on the 95 plants collected. These analyses were carried out on leaves, stems and roots, and three samples tested positive for *M*. *ulcerans* DNA by PCR: a leaf pool and a stem pool from the same plant from a water body in Kode and a leaf pool from Mitro (S4 Table). Both plants concerned belonged to the Poaceae plant family.

## Discussion

The ecological characteristics and mode of transmission of *M*. *ulcerans* are not entirely understood, and several fundamental questions remain unanswered. One key concern relates to the routes by which *M*. *ulcerans* crosses the human skin barrier. There are currently two main hypotheses: (i) direct contact between an existing wound and water containing *M*. *ulcerans*; (ii) the inoculation of *M*. *ulcerans* into the skin [2,45]. Comparisons with the modes of transmission of other environmental mycobacteria in immunocompetent humans (e.g. *M*. *fortuitum*, *M*. *chelonae*, *M*. *xenopy*) and recent studies of *M*. *ulcerans* [46] have suggested that direct inoculation into the skin is the most likely mode of transmission. In this context, the two most likely scenarios for the inoculation with the bacterium are either inoculation by an active vector harboring *M*. *ulcerans*, as described for various microorganisms, including parasites (e.g. *Leishmania* sp. or *Plasmodium* sp.), arboviruses (e.g. the Dengue and Chikungunya viruses), and bacteria (e.g. *Yersinia pestis* and *Borrelia* sp.), or inoculation by a mechanical vector, such as aquatic plant thorns or sharp leaves, biting or sucking insects (bacilli present on the outside of the insects) [13,15,16,17,18,19,20,21,25,26,27,28,29,30,47,48,49,50,51,52]. *M*. *ulcerans* ecology is highly complex. It is therefore possible for these scenarios to co-exist, and their importance or significance is dependent on a number of different criteria (e.g. human behavior, including access to drinking water, rural or urban life and work, fauna and flora biodiversity, presence of permissive species, season).

Several experimental studies have explored the role of aquatic hemipterans as passive or active vectors of *M*. *ulcerans*. These approaches were supported by various environmental and epidemiological studies conducted in Africa. However, the importance (unique, major, or marginal) of this transmission route has yet to be established and other transmission routes should therefore be explored. For instance, it has been suggested that mosquitoes act as vectors of *M*. *ulcerans* in Australia, but, surprisingly, this possibility has never been explored in Africa. In this context, the aim of our study was to assess the role of mosquitoes in *M*. *ulcerans* ecology. We carried out an extensive field study in an endemic area in Benin, involving temporal and spatial monitoring of the presence of *M*. *ulcerans* in mosquitoes and other flying insects, used as a control for the distribution of *M*. *ulcerans* in aquatic flora and fauna.

*M*. *ulcerans* DNA was detected in various aquatic macro-invertebrates and vertebrates, and some aquatic plants. The global rate of detection was about 9%, consistent with the findings of other environmental studies [26,27,28,29,30]. *M*. *ulcerans* DNA was not detected in any of the flying insects collected in CDC light traps inside and around houses over the same period (including mosquito families in which *M*. *ulcerans* DNA was detected in Australia). As only one type of sampling method was used to collect flying insects (CDC light traps), it is possible that this introduced a bias in terms of species diversity. Nevertheless, in a recent study performed in the same area with three other types of sampling method for mosquito collection, the three most abundant mosquito species were the same as in our study, and eight of the 14 species identified were common to our study [53].

Our results suggest that mosquitoes and non-aquatic flying insects are not involved in the ecology and dissemination of *M*. *ulcerans* in an area of South-East Benin in which Buruli ulcer is highly endemic, and confirm that the aquatic environment is the main environmental reservoir of the bacillus. However, a role for mosquitoes in other areas, including Australia, cannot be definitively excluded.

The ecology and mode of transmission of micro-organisms may differ between geographic locations, with biological diversity affecting bacterial adaptation and human activities. This concept could be applied to *M*. *leprae*, a mycobacterium that also causes a dermatosis. Indeed, a recent study has suggested that the ecological features, reservoirs and transmission routes of *M*. *leprae* may differ between continents. It has been shown that, in North America, wild armadillos harbor the same strain of *M*. *leprae* as leprosy patients. Leprosy may thus be a zoonosis in this region [54]. This situation cannot be transposed to other continents in which leprosy is highly endemic such as Africa and Asia, where there are no armadillos and no other mammal is known to harbor the bacillus. A similar situation may apply to *M*. *ulcerans*. In Australia, mammals such as possums have been shown to be hosts of *M*. *ulcerans* and may play a key role in its dissemination, together with mosquitoes. However, there are no possums in Africa, and *M*. *ulcerans* has never been detected in the tissues of any mammal other than humans in Africa.

Based on the results of various studies performed in recent decades and aiming to decipher the ecological characteristics of *M*. *ulcerans*, it seems likely that *M*. *ulcerans* can be transmitted via several routes, potentially differing between locations in different parts of the world.

## Supporting Information

S1 Table Mosquito species collected during the surveys. (DOCX)

S2 Table Total mosquitoes and other insects collected per site and per season. (DOCX)

S3 Table Mosquito larvae and emerged adults sampled during the surveys. (DOCX)

S4 Table Ct values for qPCR-positive samples. (DOCX)

## References

[1] Asiedu K, Etuaful S (1998) Socioeconomic implications of Buruli ulcer in Ghana: a three-year review. Am J Trop Med Hyg 59: 1015–1022. 9886216 10.4269/ajtmh.1998.59.1015

[2] Asiedu K, Sherpbier R, Raviglione MC (2000) Buruli Ulcer *Mycobacterium ulcerans* infection. W.H.O. Global Buruli Ulcer initiative. Report 2000 World Health Organisation Geneva Switzerland.

[3] Vincent QB, Ardant MF, Adeye A, Goundote A, Saint-Andre JP, Cottin J, et al (2014) Clinical epidemiology of laboratory-confirmed Buruli ulcer in Benin: a cohort study. Lancet Glob Health 2: e422–430. 10.1016/S2214-109X(14)70223-2 25103396

[4] Marion E, Song OR, Christophe T, Babonneau J, Fenistein D, Eyer J, et al (2014) Mycobacterial toxin induces analgesia in buruli ulcer by targeting the angiotensin pathways. Cell 157: 1565–1576. 10.1016/j.cell.2014.04.040 24949969

[5] Chauty A, Ardant MF, Adeye A, Euverte H, Guedenon A, Johnson C, et al (2007) Promising clinical efficacy of streptomycin-rifampin combination for treatment of buruli ulcer (*Mycobacterium ulcerans* disease). Antimicrob Agents Chemother 51: 4029–4035. 17526760 10.1128/AAC.00175-07 PMC2151409

[6] Etuaful S, Carbonnelle B, Grosset J, Lucas S, Horsfield C, Phillips R, et al (2005) Efficacy of the combination rifampin-streptomycin in preventing growth of *Mycobacterium ulcerans* in early lesions of Buruli ulcer in humans. Antimicrob Agents Chemother 49: 3182–3186. 16048922 10.1128/AAC.49.8.3182-3186.2005 PMC1196249

[7] Chauty A, Ardant MF, Marsollier L, Pluschke G, Landier J, Adeye A, et al (2011) Oral treatment for *Mycobacterium ulcerans* infection: results from a pilot study in Benin. Clin Infect Dis 52: 94–96. 10.1093/cid/ciq072 21148526

[8] World Health Organization, editor (2012) Treatment of Mycobacterium ulcerans (Buruli ulcer): Guidance for health workers. Geneva. 73 p.

[9] Carolan K, Garchitorena A, Garcia-Pena GE, Morris A, Landier J, Fontanet A, et al (2014) Topography and Land Cover of Watersheds Predicts the Distribution of the Environmental Pathogen Mycobacterium ulcerans in Aquatic Insects. PLoS Negl Trop Dis 8: e3298 10.1371/journal.pntd.0003298 25375173 PMC4222759

[10] Sopoh GE, Johnson RC, Anagonou SY, Barogui YT, Dossou AD, Houezo JG, et al (2011) Buruli ulcer prevalence and altitude, Benin. Emerg Infect Dis 17: 153–154. 10.3201/eid1701.100644 21192889 PMC3204629

[11] Doannio JM, Konan KL, Dosso FN, Kone AB, Konan YL, Sankare Y, et al (2011) [ *Micronecta sp* (Corixidae) and *Diplonychus sp* (Belostomatidae), two aquatic Hemiptera hosts and/or potential vectors of *Mycobacterium ulcerans* (pathogenic agent of Buruli ulcer) in Cote d'Ivoire]. Med Trop (Mars) 71: 53–57. 21585092

[12] Marion E, Deshayes C, Chauty A, Cassisa V, Tchibozo S, Cottin J, et al (2011) [Detection of *Mycobacterium ulcerans* DNA in water bugs collected outside the aquatic environment in Benin]. Med Trop (Mars) 71: 169–172. 21695876

[13] Marion E, Eyangoh S, Yeramian E, Doannio J, Landier J, Aubry J, et al (2010) Seasonal and regional dynamics of *M*. *ulcerans* transmission in environmental context: deciphering the role of water bugs as hosts and vectors. PLoS Negl Trop Dis 4: e731 10.1371/journal.pntd.0000731 20625552 PMC2897839

[14] Marsollier L, Severin T, Aubry J, Merritt RW, Saint Andre JP, Legras P, et al (2004) Aquatic snails, passive hosts of *Mycobacterium ulcerans* . Appl Environ Microbiol 70: 6296–6298. 15466578 10.1128/AEM.70.10.6296-6298.2004 PMC522119

[15] Carolan K, Ebong SM, Garchitorena A, Landier J, Sanhueza D, Texier G, et al (2014) Ecological niche modelling of Hemipteran insects in Cameroon; the paradox of a vector-borne transmission for Mycobacterium ulcerans, the causative agent of Buruli ulcer. Int J Health Geogr 13: 44 10.1186/1476-072X-13-44 25344052 PMC4213541

[16] Marsollier L, Deniaux E, Brodin P, Marot A, Wondje CM, Saint-Andre JP, et al (2007) Protection against *Mycobacterium ulcerans* lesion development by exposure to aquatic insect saliva. PLoS Med 4: e64 17326707 10.1371/journal.pmed.0040064 PMC1808094

[17] Marsollier L, Robert R, Aubry J, Saint Andre JP, Kouakou H, Legras P, et al (2002) Aquatic insects as a vector for *Mycobacterium ulcerans* . Appl Environ Microbiol 68: 4623–4628. 12200321 10.1128/AEM.68.9.4623-4628.2002 PMC124085

[18] Marion E, Chauty A, Yeramian E, Babonneau J, Kempf M, Marsollier L (2014) A case of guilt by association: Water Bug bite incriminated in *M*. *ulcerans* infection. Internationnal Journal of Mycobacteriology 3: 168–161. 10.1016/j.ijmyco.2014.01.004 26786340

[19] Marsollier L, Brodin P, Jackson M, Kordulakova J, Tafelmeyer P, Carbonnelle E, et al (2007) Impact of *Mycobacterium ulcerans* biofilm on transmissibility to ecological niches and Buruli ulcer pathogenesis. PLoS Pathog 3: e62 17480118 10.1371/journal.ppat.0030062 PMC1864991

[20] Marsollier L, Andre JP, Frigui W, Reysset G, Milon G, Carbonnelle B, et al (2007) Early trafficking events of *Mycobacterium ulcerans* within *Naucoris cimicoides* . Cell Microbiol 9: 347–355. 16939536 10.1111/j.1462-5822.2006.00790.x

[21] Garchitorena A, Roche B, Kamgang R, Ossomba J, Babonneau J, Landier J, et al (2014) Mycobacterium ulcerans ecological dynamics and its association with freshwater ecosystems and aquatic communities: results from a 12-month environmental survey in Cameroon. PLoS Negl Trop Dis 8: e2879 10.1371/journal.pntd.0002879 24831924 PMC4022459

[22] Portaels F, Elsen P, Guimaraes-Peres A, Fonteyne PA, Meyers WM (1999) Insects in the transmission of *Mycobacterium ulcerans* infection. Lancet 353: 986 10459918 10.1016/S0140-6736(98)05177-0

[23] Portaels F, Meyers WM, Ablordey A, Castro AG, Chemlal K, de Rijk P, et al (2008) First cultivation and characterization of *Mycobacterium ulcerans* from the environment. PLoS Negl Trop Dis 2: e178 10.1371/journal.pntd.0000178 18365032 PMC2268003

[24] Kenu E, Nyarko KM, Seefeld L, Ganu V, Kaser M, Lartey M, et al (2014) Risk factors for buruli ulcer in Ghana-a case control study in the Suhum-Kraboa-Coaltar and Akuapem South Districts of the eastern region. PLoS Negl Trop Dis 8: e3279 10.1371/journal.pntd.0003279 25411974 PMC4238991

[25] Mosi L, Williamson H, Wallace JR, Merritt RW, Small PL (2008) Persistent association of Mycobacterium ulcerans with the West African predaceous insect, Belostomatidae. Appl Environ Microbiol. 10.1128/AEM.01234-08 PMC2583505 18836026

[26] Johnson PD, Azuolas J, Lavender CJ, Wishart E, Stinear TP, Hayman JA, et al (2007) Mycobacterium ulcerans in mosquitoes captured during outbreak of Buruli ulcer, southeastern Australia. Emerg Infect Dis 13: 1653–1660. 10.3201/eid1311.061369 18217547 PMC3375796

[27] Quek TY, Athan E, Henry MJ, Pasco JA, Redden-Hoare J, Hughes A, et al (2007) Risk factors for Mycobacterium ulcerans infection, southeastern Australia. Emerg Infect Dis 13: 1661–1666. 10.3201/eid1311.061206 18217548 PMC3375781

[28] Tobias NJ, Seemann T, Pidot SJ, Porter JL, Marsollier L, Marion E, et al (2009) Mycolactone gene expression is controlled by strong SigA-like promoters with utility in studies of *Mycobacterium ulcerans* and buruli ulcer. PLoS Negl Trop Dis 3: e553 10.1371/journal.pntd.0000553 19936295 PMC2775157

[29] Wallace JR, Gordon MC, Hartsell L, Mosi L, Benbow ME, Merritt RW, et al (2010) Interaction of *Mycobacterium ulcerans* with mosquito species: implications for transmission and trophic relationships. Appl Environ Microbiol 76: 6215–6222. 10.1128/AEM.00340-10 20675453 PMC2937476

[30] Lavender CJ, Fyfe JA, Azuolas J, Brown K, Evans RN, Ray LR, et al (2011) Risk of Buruli ulcer and detection of *Mycobacterium ulcerans* in mosquitoes in southeastern Australia. PLoS Negl Trop Dis 5: e1305 10.1371/journal.pntd.0001305 21949891 PMC3176747

[31] Debacker M, Aguiar J, Steunou C, Zinsou C, Meyers WM, Scott JT, et al (2004) Mycobacterium ulcerans disease: role of age and gender in incidence and morbidity. Trop Med Int Health 9: 1297–1304. 15598261 10.1111/j.1365-3156.2004.01339.x

[32] Muelder K, Nourou A (1990) Buruli ulcer in Benin. Lancet 336: 1109–1111. 1977990 10.1016/0140-6736(90)92581-2

[33] Lagarrigue V, Portaels F, Meyers WM, Aguiar J (2000) [Buruli ulcer: risk of bone involvement! Apropos of 33 cases observed in Benin]. Med Trop (Mars) 60: 262–266. 11258059

[34] Debacker M, Aguiar J, Steunou C, Zinsou C, Meyers WM, Guedenon A, et al (2004) Mycobacterium ulcerans disease (Buruli ulcer) in rural hospital, Southern Benin, 1997–2001. Emerg Infect Dis 10: 1391–1398. 15496239 10.3201/eid1008.030886 PMC3320395

[35] Debacker M, Aguiar J, Steunou C, Zinsou C, Meyers WM, Portaels F (2005) Buruli ulcer recurrence, Benin. Emerg Infect Dis 11: 584–589. 15829198 10.3201/eid1104.041000 PMC3320346

[36] Johnson RC, Makoutode M, Sopoh GE, Elsen P, Gbovi J, Pouteau LH, et al (2005) Buruli ulcer distribution in Benin. Emerg Infect Dis 11: 500–501. 15789490 10.3201/eid1103.040597 PMC3298242

[37] Gillies M, De Meillon B (1968) The Anophelinae of Africa south of the Sahara. The South African Institute for Medical Research 54.

[38] Edwards F (1941) Mosquitoes of the Ethiopian Region III. Culicine adults and pupae. In: Hist.) BMN, editor. London.

[39] Robert V, Awono-Ambene HP, Thioulouse J (1998) Ecology of larval mosquitoes, with special reference to Anopheles arabiensis (Diptera: Culcidae) in market-garden wells in urban Dakar, Senegal. J Med Entomol 35: 948–955. 9835685 10.1093/jmedent/35.6.948

[40] De Moor IJ, Day AJ, De Moor CF (2003) Guideto the Freshwater Invertebrates of Southern Africa. 288 p.

[41] Durinck S, Allemeersch J, Carey VJ, Moreau Y, De Moor B (2004) Importing MAGE-ML format microarray data into BioConductor. Bioinformatics 20: 3641–3642. 15256416 10.1093/bioinformatics/bth396

[42] Stinear TP, Mve-Obiang A, Small PL, Frigui W, Pryor MJ, Brosch R, et al (2004) Giant plasmid-encoded polyketide synthases produce the macrolide toxin of *Mycobacterium ulcerans* . Proc Natl Acad Sci U S A 101: 1345–1349. 14736915 10.1073/pnas.0305877101 PMC337055

[43] Stinear TP, Pryor MJ, Porter JL, Cole ST (2005) Functional analysis and annotation of the virulence plasmid pMUM001 from *Mycobacterium ulcerans* . Microbiology 151: 683–692. 15758215 10.1099/mic.0.27674-0

[44] Babonneau J, Marion E, Robert R, Marsollier L (in press) Development of a Dry-Reagent-Based qPCR to Facilitate the Diagnosis of Mycobacterium ulcerans Infection in Endemic Countries. PLoS Negl Trop Dis. 10.1371/journal.pntd.0003606 PMC4382021 25830546

[45] Ross BC, Johnson PD, Oppedisano F, Marino L, Sievers A, Stinear T, et al (1997) Detection of *Mycobacterium ulcerans* in environmental samples during an outbreak of ulcerative disease. Appl Environ Microbiol 63: 4135–4138. 9327583 10.1128/aem.63.10.4135-4138.1997 PMC168730

[46] Williamson HR, Mosi L, Donnell R, Aqqad M, Merritt RW, Small PL (2014) Mycobacterium ulcerans fails to infect through skin abrasions in a guinea pig infection model: implications for transmission. PLoS Negl Trop Dis 8: e2770 10.1371/journal.pntd.0002770 24722416 PMC3983084

[47] UgandaBuruliGroup (1971) Epidemiology of Mycobacterium ulcerans infection (Buruli ulcer) at Kinyara, Uganda. Trans R Soc Trop Med Hyg 65: 763–775. 5157438 10.1016/0035-9203(71)90090-3

[48] Marion E, Landier J, Boisier P, Marsollier L, Fontanet A, Le Gall P, et al (2011) Geographic expansion of Buruli ulcer disease, Cameroon. Emerg Infect Dis 17: 551–553. 10.3201/eid1703.091859 21392458 PMC3165989

[49] Marsollier L, Aubry J, Coutanceau E, Andre JP, Small PL, Milon G, et al (2005) Colonization of the salivary glands of *Naucoris cimicoides* by *Mycobacterium ulcerans* requires host plasmatocytes and a macrolide toxin, mycolactone. Cell Microbiol 7: 935–943. 15953026 10.1111/j.1462-5822.2005.00521.x

[50] Marsollier L, Stinear T, Aubry J, Saint Andre JP, Robert R, Legras P, et al (2004) Aquatic plants stimulate the growth of and biofilm formation by *Mycobacterium ulcerans* in axenic culture and harbor these bacteria in the environment. Appl Environ Microbiol 70: 1097–1103. 14766593 10.1128/AEM.70.2.1097-1103.2004 PMC348869

[51] McIntosh M, Williamson H, Benbow ME, Kimbirauskas R, Quaye C, Boakye D, et al (2014) Associations between Mycobacterium ulcerans and aquatic plant communities of West Africa: implications for Buruli ulcer disease. Ecohealth 11: 184–196. 10.1007/s10393-013-0898-3 24442959

[52] Merritt RW, Walker ED, Small PL, Wallace JR, Johnson PD, Benbow ME, et al (2010) Ecology and transmission of Buruli ulcer disease: a systematic review. PLoS Negl Trop Dis 4: e911 10.1371/journal.pntd.0000911 21179505 PMC3001905

[53] Padonou GG, Sezonlin M, Gbedjissi G, Ayi I, Azondekon R, Djenontin A, et al (2011) Biology of Anopheles gambiae and insecticide resistance: Entomological study for a large scale of indoor residual spraying in South East Benin. J Parasitol Vector Biol 3: 59–68.

[54] Truman RW, Singh P, Sharma R, Busso P, Rougemont J, Paniz-Mondolfi A, et al (2011) Probable zoonotic leprosy in the southern United States. N Engl J Med 364: 1626–1633. 10.1056/NEJMoa1010536 21524213 PMC3138484

