# Supplementary material for: A Field Study in Benin to Investigate the Role of Mosquitoes and Other Flying Insects in the Ecology of Mycobacterium ulcerans
Source: PLoS Negl Trop Dis. 2015 Jul 21;9(7):e0003941. doi: 10.1371/journal.pntd.0003941 (PMC4510061; doi:10.1371/journal.pntd.0003941)
Supplement: S1 Table — (DOCX) [file pntd.0003941.s001.docx]

**Table S1: Mosquito species collected during the surveys**

|  |  |  |  |  |  |  | |  | |  | |  | |  |  |  |  |  |  | |  |  |  |  |
| --- | --- | --- | --- | --- | --- | --- | --- | --- | --- | --- | --- | --- | --- | --- | --- | --- | --- | --- | --- | --- | --- | --- | --- | --- |
|  | Survey 1 | | | | Survey 2 | | | | | | | | Survey 3 | | | | | Survey 4 | | | | | | Total |
|  | Gbada | Bonou | Houeda | Kode | Gbada | | Bonou | | Houeda | | Kode | | Gbada | | Bonou | Houeda | Kode | Gbada | | Bonou | | Houeda | Kode |  |
| *Anopheles gambiae* | 0 | 9 | 14 | 8 | 0 | | 2 | | 3 | | 1 | | 0 | | 4 | 1 | 2 | 0 | | 1 | | 0 | 2 | 47 |
| *Anopheles ziemanni* | 0 | 4 | 5 | 0 | 0 | | 0 | | 2 | | 0 | | 2 | | 0 | 3 | 0 | 0 | | 0 | | 2 | 1 | 19 |
| *Anopheles pharoensis* | 1 | 0 | 3 | 0 | 0 | | 0 | | 0 | | 0 | | 0 | | 0 | 0 | 0 | 0 | | 0 | | 0 | 0 | 4 |
| *Aedes luteocephalus* | 1 | 0 | 0 | 1 | 8 | | 5 | | 0 | | 5 | | 0 | | 0 | 0 | 0 | 2 | | 0 | | 0 | 0 | 22 |
| *Aedes vittatus* | 0 | 0 | 0 | 1 | 0 | | 0 | | 0 | | 0 | | 0 | | 0 | 0 | 0 | 0 | | 0 | | 0 | 0 | 1 |
| *Culex decens* | 0 | 3 | 0 | 0 | 0 | | 0 | | 0 | | 0 | | 0 | | 0 | 0 | 1 | 0 | | 0 | | 0 | 0 | 4 |
| *Culex fatigans* | 0 | 0 | 1 | 0 | 0 | | 0 | | 0 | | 0 | | 1 | | 0 | 0 | 1 | 0 | | 0 | | 0 | 0 | 3 |
| *Culex nebulosus* | 136 | 62 | 44 | 82 | 1 | | 15 | | 22 | | 65 | | 115 | | 29 | 122 | 119 | 9 | | 8 | | 30 | 264 | 1123 |
| *Culex quinquefasciatus* | 32 | 213 | 110 | 230 | 28 | | 8 | | 86 | | 18 | | 24 | | 23 | 12 | 84 | 20 | | 11 | | 10 | 31 | 940 |
| *Mansonia africana* | 16 | 87 | 175 | 212 | 10 | | 17 | | 160 | | 94 | | 277 | | 93 | 373 | 145 | 174 | | 194 | | 20 | 112 | 2159 |
| Total | 186 | 378 | 352 | 534 | 47 | | 47 | | 273 | | 183 | | 419 | | 149 | 511 | 352 | 205 | | 214 | | 62 | 410 | 4322 |
