# Supplementary material for: A Field Study in Benin to Investigate the Role of Mosquitoes and Other Flying Insects in the Ecology of Mycobacterium ulcerans
Source: PLoS Negl Trop Dis. 2015 Jul 21;9(7):e0003941. doi: 10.1371/journal.pntd.0003941 (PMC4510061; doi:10.1371/journal.pntd.0003941)
Supplement: S2 Table — (DOCX) [file pntd.0003941.s002.docx]

**Table S2: Total mosquitoes and other insects collected per site and per season**

|  | | Mosquitoes | Other insects |
| --- | --- | --- | --- |
| Sites | Gbada | 857 | 697 |
|  | Bonou | 788 | 718 |
|  | Houeda | 1198 | 1068 |
|  | Kode | 1479 | 1027 |
|  | *p* | >0.05 | >0.05 |
| Seasons | Rainy season | 2000 | 1649 |
|  | Dry season | 2322 | 1861 |
|  | *p* | >0.05 | <0.05 |
