# Supplementary material for: A Field Study in Benin to Investigate the Role of Mosquitoes and Other Flying Insects in the Ecology of Mycobacterium ulcerans
Source: PLoS Negl Trop Dis. 2015 Jul 21;9(7):e0003941. doi: 10.1371/journal.pntd.0003941 (PMC4510061; doi:10.1371/journal.pntd.0003941)
Supplement: S3 Table — (DOCX) [file pntd.0003941.s003.docx]

**Table S3: Mosquito larvae and emerged adults sampled during the surveys**

|  | Larvae (Group 1) | | | | Emerged adults (Group 2) | | | | Total |
| --- | --- | --- | --- | --- | --- | --- | --- | --- | --- |
|  | Gbada | Bonou | Houeda | Kode | Gbada | Bonou | Houeda | Kode |  |
| *Culex* spp. | 784 | 392 | 676 | 235 | 615 | *410* | 450 | 313 | 3875 |
| *Anopheles* spp. | 20 | 399 | 20 | 620 | 0 | *198* | 31 | 197 | 1485 |
| *Aedes* spp. | 0 | 0 | 0 | 0 | 0 | *2* | 45 | 0 | 47 |
| Total | 804 | 791 | 696 | 855 | 615 | *610* | 526 | 510 | 5407 |
