# Supplementary material for: A Field Study in Benin to Investigate the Role of Mosquitoes and Other Flying Insects in the Ecology of Mycobacterium ulcerans
Source: PLoS Negl Trop Dis. 2015 Jul 21;9(7):e0003941. doi: 10.1371/journal.pntd.0003941 (PMC4510061; doi:10.1371/journal.pntd.0003941)
Supplement: S4 Table — (DOCX) [file pntd.0003941.s004.docx]

**Table S4: Ct values for qPCR-positive samples**

| Type | Site | Sample | Ct KR | Ct IS2404 |
| --- | --- | --- | --- | --- |
| Aquatic animals | Mitro | Coleoptera | 34.15 | 32.2 |
|  | Mitro | Hemiptera | 32.06 | 30.54 |
|  | Mitro | Coleoptera | 33.87 | 32.97 |
|  | Mitro | Hemiptera | 30.3 | 27.59 |
|  | Houeda | Coleoptera | 33.02 | 32.12 |
|  | Houeda | Anura | 31.71 | 29.75 |
|  | Houeda | Coleoptera | 28.71 | 26.62 |
|  | Houeda | Hemiptera | 31.68 | 29.61 |
|  | Agonhoui | Decapoda | 29.1 | 27.07 |
|  | Agonhoui | Hemiptera | 34.93 | 33.98 |
|  | Agonhoui | Hemiptera | 29.03 | 27.25 |
|  | Agonhoui | Odonota | 34.77 | 33.21 |
|  | Agonhoui | Decapoda | 34.77 | 33.21 |
|  | Agbonan | Diptera | 34.89 | 34.88 |
|  | Agbonan | Hemiptera | 33.8 | 31.2 |
|  | Assigui | Anura | 30.82 | 28.97 |
|  | Assigui | Hemiptera | 33.12 | 31.32 |
|  | Assigui | Hemiptera | 32.96 | 31.11 |
|  | Assigui | Fish | 28.22 | 26.83 |
|  | Assigui | Decapoda | 29.64 | 29.62 |
|  | Assigui | Decapoda | 35.2 | 33.15 |
|  | Assigui | Decapoda | 32.9 | 31.48 |
|  | Assigui | Decapoda | 34.18 | 31.25 |
|  | Assigui | Hemiptera | 33.95 | 33.55 |
|  | Assigui | Decapoda | 30.11 | 30.38 |
|  | Kode | Hemiptera | 34.57 | 32.84 |
|  | Kode | Coleoptera | 28.82 | 27.23 |
|  | Bonou | Coleoptera | 34.33 | 33.86 |
| Aquatic plants | Kode | leaf | 31.09 | 30.17 |
|  | Kode | stem | 27.69 | 25.23 |
|  | Mitro | leaf | 33.09 | 31.48 |
